# The Role of Race and Economic Characteristics in the Presentation and Survival of Patients With Surgically Resected Non-Small Cell Lung Cancer

**DOI:** 10.3389/fonc.2018.00146

**Published:** 2018-05-14

**Authors:** John M. Varlotto, Kerri McKie, Rickie P. Voland, John C. Flickinger, Malcolm M. DeCamp, Debra Maddox, Paul Stephen Rava, Thomas J. Fitzgerald, William Walsh, Paulo Oliveira, Negar Rassaei, Jennifer Baima, Karl Uy

**Affiliations:** ^1^Department of Radiation Oncology, University of Massachusetts Medical Center, Worcester, MA, United States; ^2^University of Massachusetts Medical School, Worcester, MA, United States; ^3^School of Nursing, University of Wisconsin, Madison, WI, United States; ^4^Department of Radiation Oncology, University of Pittsburgh Medical Center, Pittsburgh, PA, United States; ^5^Division of Thoracic Surgery, Northwestern Memorial Medical Center, Chicago, IL, United States; ^6^Department of Medical Oncology, University of Massachusetts Medical Center, Worcester, MA, United States; ^7^Division of Pulmonary, Allergy and Critical Care Medicine, Worcester, MA, United States; ^8^Department of Pathology, Penn State Hershey Medical Center, Hershey, PA, United States; ^9^Division of Physical Medicine and Rehabilitation, Worcester, MA, United States; ^10^Division of Thoracic Surgery, University of Massachusetts Medical Center, Worcester, MA, United States

**Keywords:** lung cancer, surgical resection, socioeconomic status, marital status, racial differences

## Abstract

**Background:**

Little is understood regarding the inter-relation between economic, marital, and racial/ethnic differences in presentation and survival of surgically resected lung cancer patients. Our investigation will assess these differences in addition to known therapeutic, patient, and histopathologic factors.

**Methods:**

A retrospective review of the Surveillance Epidemiology and End Reporting database was conducted through the years 2007–2012. The population was split into nine different ethnic groups. Population differences were assessed *via* chi-square testing. Multivariable analysis (MVA) were used to detect overall survival (OS) differences in the total surgical population (TS, *N* = 35,689) in an ear (T1–T2 < 4 cm N0) surgical population [early-stage resectable (ESR), *N* = 17,931]. Lung cancer-specific survival (LCSS) was assessed in the ESR.

**Results:**

In the TS population, as compared to Whites, Blacks, and Hispanics presented with younger age, more adenocarcinomas, lower rates of marriage, lower rates of insurance, less stage I tumors, and had less nodes examined, but their type of surgical procedures and OS/LCSS were the same. MVA demonstrated that lower OS and LCSS were associated with males, single/divorced/widowed partnership, lower income (TS only), and Medicaid insurance. MVA also found that Blacks and Hispanics had a similar OS/LCSS to Whites and that all ethnic groups were associated with a similar or better outcomes. The 90-day mortality and positive nodes were correlated with not having insurance and not being married, but they were not associated with ethnicity.

**Conclusion:**

In TS and ESR groups, OS was not different in the two largest ethnic groups (Black and Hispanic) as compared to Whites, but was related to single/widowed/divorced status, Medicaid insurance, and income (TS group only). Nodal positivity was associated with patients who did not have a married partner or insurance suggesting that these factors may impact disease biology. Economic and psychosocial variables may play a role in survival of ear lung cancer in addition to standard histopathologic and treatment variables.

## Introduction

Surgery is the standard treatment option for patients with early-stage, medically operable patients because of its known long-term efficacy (1).

The relationship between patients chosen for surgical therapy and their outcome in relation to economic, insurance, partnership, and racial issues has been infrequently studied. A recent retrospective study using the VA Central Cancer Registry in stage I/II non-small cell lung cancer (NSCLC) from 2001 to 2010 demonstrated that the disparity between Blacks and Whites receiving an operation decreased to similar rates during this time period. Furthermore, there was no survival difference between Black and Whites undergoing an operation, and no lung cancer-specific survival (LCSS) differences between races (2). Using data compiled from 38 state and the District of Columbia population-based cancer registries compiled by the North American Association of Central Cancer Registries, Sineshaw et al. demonstrated that the receipt of curative-intent surgery varied by state and was lower in blacks than whites in every state (statistically significant in Texas and Florida) (3). Similarly, using the Surveillance Epidemiology and End Reporting (SEER) database from 2007 to 2012, Taioli and Flores noted that even after adjusting by age and insurance status, blacks were less likely to receive surgery, but more likely to receive radiation than white patients (4). However, none of these studies evaluate race in relation to economic, marital, and insurance variables. Nor have these reports analyzed differences in outcome in the many different ethnic groups who are found in the United States.

Because lung cancer screening was shown to be of benefit in 2011 (5) and was approved by CMS in 2015, early-stage resectable (ESR) NSCLC is expected to increase and result in more lung cancer survivors (6). Therefore, assessing the presentation and outcomes of patients undergoing surgery for NSCLC and inter-relationship of ethnicity in regards to marital, economic, histologic, treatment, and insurance variables will be increasingly important.

The purpose of our study is to investigate the presenting characteristics of patients undergoing a definitive surgical procedure in nine different ethnic groups [White non-Hispanic (White), Black, White Hispanic (Hispanic), American Indian/Alaskan native (AI/AN), Chinese, Japanese, Other Asian, South Asian, and Other Race] and to assess prognosis and 90-day mortality for all surgical patients and for those presenting with early-stage, resectable tumors (ESR, <4 cm without involved nodes). The prognostic importance of race will be determined in a multivariate model that adjusts for known histopathologic and patient-related factors as well as income, marital status, and insurance.

## Materials and Methods

### Data Source

Data for this study were taken from the SEER program of the National Cancer Institute, which started to collect and publish cancer incidence and survival data from population-based cancer registries in 1973. The “SEER-18” database used in this study includes registries in Atlanta, Connecticut, Detroit, Hawaii, Iowa, New Mexico, San Francisco-Oakland, Seattle-Puget Sound, Utah, Los Angeles, San Jose-Monterey, Rural Georgia, Greater California, Kentucky, Louisiana, New Jersey, Greater Georgia, and the Alaska Native Tumor Registry (7). Data are available from all cases diagnosed from 2000 and later for these registries. The SEER 18 sites cover approximately 28% of the American population (7).

### Cohort Selection

We included adults, ages who were at least 18 years old and who were diagnosed with histologically proven NSCLC in the SEER-18 database during 2007–2012.

Outcome and presenting characteristics were examined for all surgical patients (TS) (*N* = 35,689) and patients with presenting with ESR disease (*N* = 17,931) for whom sufficient information was collected to assess the outcome of treatment in relation to patient, economic, histopathologic, and insurance variables. Patients included in this investigation had NSCLC as their first primary cancer. Only microscopically confirmed tumors using NSCLC codes (8012-8014,8022,8031-8033,8046,8052,8070-8073,8082,8084,8123,8140,8200,8230,8250-8255,8260,8310,8333,8430,8470,8480-8481,8490,8550,8560,8972,8980) were included in this study.

Only patients undergoing a definitive surgical procedure without pre-operative radiation were included in this analysis. The surgical procedures defined as definitive were as follows: sublobar resection (sublobar resection; segmental resection, including lingulectomy; or wedge resection); and lobectomy or greater (lobectomy or bi-lobectomy, with or without extension to include the chest wall; lobectomy with mediastinal node dissection; extended lobectomy or bi-lobectomy, not otherwise specified; pneumonectomy with mediastinal node dissection; or pneumonectomy, not otherwise specified).

### Outcome Variables and Other Covariates

The outcome variables were overall survival (OS) and LCSS. Deaths from other causes were treated as censoring events. The main purpose of our investigation was to examine whether there are differences in presenting characteristics and outcomes in nine different ethnic groups by examining marital status, household income (<$50,000; $50–$74,999; >$75,000), type of insurance (insured, Medicaid, uninsured, unknown) in addition to established histopathologic and patient factors. Household income was listed in the SEER registry by median household income per county. The population was split into nine different ethnic groups as follows: White non-Hispanic (White), Black, White Hispanic (Hispanic), AI/AN, Chinese, Japanese, South Asian (Asian Indian and Pakistani), Other Asian (Filipino, Thai, Vietnamese, Korean, Kampuchean, Laotian, and Hmong), and Other Race (OR, Chamorran, Fiji Islander, Guamanian, Hawaiian, Melanesian, Micronesian, New Guinean, Pacific Islander, Polynesian, Samoan, Tahitian, Tongan, unknown, and other) in both the entire lung cancer surgical population as well as those presenting with ESR disease. We originally wanted to include black Hispanic patients as a separate patient category in this manuscript and its companion study assessing ethnic differences in all lung cancer patients and those with Stage IV disease, but since we wanted similar populations in both studies and because the number of Black Hispanic patients was scant in both the TS population and the ESR groups, we decided to include Black Hispanic patients in the Black category, similar to a past study (8). Black Hispanic patients represented approximately 0.6% of patient group undergoing surgical resection (19/3,276). Throughout this manuscript, the term population(s) will refer to total population of surgical patients (TS) and those with ESR disease, while group(s) will refer to the nine different ethnicities.

Variables examined for their potential effect on outcome were gender; age; year of diagnosis; marital status; race; ethnicity; tumor stage; *t*-stage, *n*-stage; nodes examined; nodes positive; node density (number of nodes positive/number of nodes examined); tumor size; histology; grade; SEER registry location; median family income; resection type; post-operative radiation; and tumor location. Median follow-up time was calculated by the methods of Schemper and Smith in which death becomes a censored follow-up time and was noted to be 36 and 35 months in the TS and ESR groups, respectively (9).

### Statistical Analysis

Chi-square and *t*-test were used to compare difference between the ethnic groups with respect to treatment, patient characteristics, and tumor characteristics. Cox proportional hazards models estimates (10) were used to calculate adjusted hazard ratios with their 95% confidence intervals, and to show how treatment and other covariates were related to OS and LCSS. Medicare eligibility was controlled through use of two strata for age at diagnosis (≥65 vs <65 years old) because individual cases will change when they enroll in Medicare. The cox proportional hazards assumption was checked by visual examination of survival plots.

## Results

### Presenting Characteristics

Complete demographic and histologic details of the TS and ESR patients can be seen in Table 1. Median age of the patients in the TS and ESR populations were both 68.0 years. There was a female predominance to both populations (50.4%—TS and 55.1%—ESR). The three largest ethnic groups in the TS were White, Black, and Hispanic, and they represented 78.6, 9.2, and 5.0% of the population, respectively. Likewise, the ESR population’s three largest ethnic groups were White (79.6%), Black (8.4%), and Hispanic (4.6%). A similar proportion of patients presented with a low median family income (<$50,000) and was noted to be 29.8 and 28.7% in the TS and ESR populations, respectively. The majority of patients were married, 57.3% (TS) and 56.2% (ESR). 87.4% (TS) and 88.3% (ESR) patients were insured. Adenocarcinoma was the predominant histology (61.7%—TS and 67.1%—ESR).

**Table 1:** Demographic characteristics of both the TS and early-stage resectable patients.

	All surgical (*N* **=** 35,689)	Favorable (*N* **=** 17,931)
**Age—years**		
Median	68.0	68.0

**Sex—no. (%**)		
Female	(50.4%) 17,989	(55.1%) 9,882
Male	(49.6%) 17,700	(44.9%) 8,693

**Race—no. (%**)		
White Hispanic	(4.98%) 1,779	(4.60%) 823
White non-Hispanic	(78.60%) 28,052	(79.60%) 14,273
Black	(9.18%) 3,276	(8.42%) 1,509
Chinese	(1.59%) 568	(1.61%) 288
Japanese	(0.85%) 302	(0.78%) 139
South Asian	(0.31%) 112	(0.31%) 56
Other Asian	(2.83%) 1,011	(2.96%) 531
Other Race	(1.28%) 457	(1.41%) 252
American Indian/Alaskan Native	(0.37%) 132	(0.33%) 60

**Surveillance Epidemiology and End Reporting Registry—no. (%)**		
Alaska Natives	(0.10%) 35	(0.07%) 13
Atlanta	(3.05%) 1,090	(2.79%) 501
California excl SF/SJM/LA	(19.24%) 6,865	(19.63%) 3,521
Connecticut	(5.99%) 2,138	(6.44%) 1,155
Detroit	(6.23%) 2,223	(6.40%) 1,148
Greater Georgia	(9.45%) 3,374	(9.46%) 1,696
Hawaii	(1.54%) 549	(1.56%) 279
Iowa	(4.32%) 1,544	(4.10%) 736
Kentucky	(9.90%) 3,534	(9.56%) 1,715
Los Angeles	(7.64%) 2,728	(7.38%) 1,324
Louisiana	(5.54%) 1,977	(5.15%) 924
New Jersey	(13.34%) 4,760	(13.92%) 2,496
New Mexico	(1.41%) 503	(1.42%) 254
Rural Georgia	(0.23%) 83	(0.23%) 42
San Francisco-Oakland	(4.47%) 1,594	(4.51%) 809
San Jose-Monterey	(2.14%) 762	(1.94%) 347
Seattle	(4.08%) 1,457	(4.34%) 779
Utah	(1.33%) 473	(1.07%) 192

**Income—no. (%)**		
<50k	(29.84%) 10,649	(28.67%) 5,140
50k–74k	(52.88%) 18,871	(53.40%) 9,575
≥75k	(17.29%) 6,169	(17.94%) 3,216

**Marital status—no. (%)**		
Divorced	(12.13%) 4,330	(12.06%) 2,162
Married	(57.33%) 20,460	(56.19%) 10,076
Separated	(0.98%) 350	(0.91%) 163
Single	(11.23%) 4,009	(10.75%) 1,927
Unknown	(3.52%) 1,258	(3.89%) 698
Domestic partner	(0.09%) 33	(0.06%) 11
Widowed	(14.71%) 5,249	(16.14%) 2,894

**AJCC T 6th edition—no. (%)**		
T0	(0.03%) 9	0
T1	(41.75%) 14,900	(69.25%) 12,417
T2	(42.36%) 15,119	(30.75%) 5,514
T3	(5.63%) 2,010	0
T4	(9.91%) 3,537	0
TX	(0.32%) 114	0

**Insurance—no. (%)**		
Insured	(87.45%) 31,210	(88.26%) 15,826
Medicaid	(9.85%) 3,516	(9.48%) 1,700
Uninsured	(2.07%) 740	(1.63%) 292
Unknown	(0.62%) 223	(0.63%) 113

**Lateral location—no. (%)**		
Bronchus, left	(0.38%) 136	(0.09%) 16
Bronchus, right	(0.32%) 116	(0.06%) 11
Bronchus, unknown	(0.03%) 9	(0.02%) 3
Left lower	(13.95%) 4,980	(13.76%) 2,467
Left upper	(26.30%) 9,388	(26.14%) 4,687
Left NOS	(0.69%) 248	(0.31%) 56
Left overlapping	(0.36%) 127	(0.12%) 22
Lung, NOS	(0.22%) 80	0
Right lower	(17.45%) 6,228	(17.30%) 3,102
Right middle	(5.00%) 1,786	(5.50%) 986
Right upper	(32.99%) 11,774	(35.71%) 6,404
Right NOS	(1.14%) 407	(0.45%) 81
Right overlapping	(1.15%) 410	(0.54%) 96

**Histology—no. (%)**		
Adenocarcinoma	(61.74%) 22,037	(67.12%) 12,036
Adenosquamous	(2.86%) 1,021	(2.44%) 438
Large cell	(3.01%) 1,075	(2.53%) 454
Non-small cell	(3.72%) 1,327	(2.76%) 495
Other	(0.99%) 355	(0.73%) 131
Squamous	(27.67%) 9,874	(24.41%) 4,377

**Grade—no. (%)**		
Moderately, II	(41.20%) 14,703	(45.29%) 8,121
Poorly, III	(36.31%) 12,960	(28.56%) 5,121
Undifferentiated, IV	(1.96%) 701	(1.39%) 250
Unknown	(6.94%) 2,476	(5.64%) 1,012
Well, I	(13.59%) 4,849	(19.11%) 3,427

**Surgical Procedure—no. (%)**		
(Bi)Lobectomy	(76.16%) 27,182	(76.79%) 13,769
Penumonectomy	(5.52%) 1,971	(0.94%) 169
Segmentectomy	(3.04%) 1,084	(4.06%) 728
Sub-lobar resection, NOS	(0.62%) 222	(0.41%) 74
Wedge	(14.65%) 5,230	(17.80%) 3,191

**Radiation—no. (%)**		
No	(85.23%) 30,419	(96.77%) 17,352
Yes	(14.77%) 5,270	(3.23%) 579

**Year of diagnosis—no. (%)**	
2007	(17.03%) 6,077	(16.81%) 3,015
2008	(17.10%) 6,103	(16.90%) 3,030
2009	(16.99%) 6,062	(17.22%) 3,087
2010	(16.67%) 5,949	(16.83%) 3,018
2011	(16.44%) 5,868	(16.63%) 2,982
2012	(15.78%) 5,630	(15.61%) 2,799

### Univariate Analysis of All Patients Undergoing Surgical Resection of Lung Cancer

**Figure 1:**
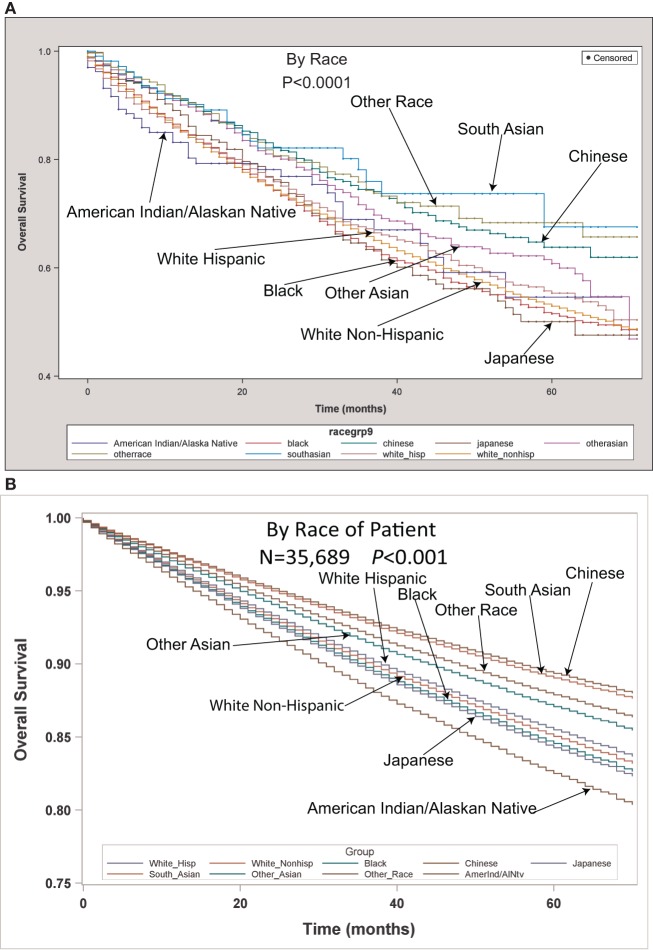
**(A)** Unadjusted overall survival (OS) by ethnic group in the TS population. **(B)** Multivariable adjusted OS by ethnic group in the TS population.

Table 2 contains the demographic, histologic, and treatment details for the TS population for the nine different ethnic groups and used the White population as the reference group. Blacks presented with a younger age, less stage I tumors, less grade I tumors, lower income, higher percentage of adenocarcinomas, less nodes examined, and were less likely to be insured, but their number of nodes positive, nodal density, OS, and LCSS was the same. Their 30 and 90-day mortality did not differ as compared to Whites. Hispanic patients presented with younger age, higher median household income, lower rates of insurance, higher percentage of females, lower percentage of Stage I, more grade 1 tumors, higher percentage of adenocarcinomas, and had less nodes examined, but they had a similar number of nodes positive, nodal density, OS and LCSS. Hispanics had a similar 90-day mortality, but their 30-day mortality was higher than Whites (mean 1.8 vs 1.1%). Of all the ethnic groups, the Japanese presented with a highest mean age (70.9), the highest female predominance (62.3%), and the highest rates of insurance (98.0%), but there was a similar OS and LCSS to Whites. Blacks (58.3%) and Hispanics (59.2%) presented with a lower proportion of patients with Stage I NSCLC as compared to Whites (63.2%), but similar rates were noted in all other ethnic groups. The Other Asian group presented with the highest percentage of adenocarcinomas (78.5%), while American/Alaskan Natives presented with the highest percentage of squamous cell carcinomas (35.6%). The Chinese had the highest proportion of patients receiving a (bi)lobectomy at 86.1%, but the least receiving a pneumonectomy (2.5%) as well as a wedge resection (8.8%). Likewise, the Chinese were least likely to undergo a sub-lobar resection for tumors greater than 2 cm with only 5.0% receiving such treatment. Blacks (8.2), Hispanics (8.5), and Other Asians (8.3) were found to have less mean nodes examined than Whites (9.0), and a higher proportion of patients with positive nodes was noted in the Other Asian group (26 vs 21.8%), but none of the other ethnic groups differed from Whites in terms of the median number of nodes explored or number of nodes positive. The only ethnic group that differed from Whites in regards to nodal density was the Other Asian group, 0.10-Other Asians vs 0.07-Whites. The 30-day mortality was higher in the Hispanic patients, but lower in the Other Race and Japanese ethnic groups. The 90-day survival was significantly higher in the Other Race and Other Asian groups. As compared to Whites, OS and LCSS was significantly greater in the Chinese, South Asian, Other Asian, and the Other Race groups. Unadjusted OS by ethnic group can be found in the Kaplan–Meier survival in Figure 1A.

**Table 2:** Demographic, histologic, and treatment details in the TS population for the nine different ethnic groups.

*N* = 35,689	White non-Hispanic	White Hispanic	Black	Chinese	Japanese	South Asian	Other Asian	Other Race	American Indian/Alaskan
Patient numbers	28,052	1,779	3,276	568	302	112	1,011	457	132
**Age at diagnosis, years**									
Mean (95% CI)	67.4 (67.3–67.5)	**66.1 (65.5–66.6)**	**63.2 (62.8–63.5)**	66.9 (66.0–67.8)	**70.9 (69.7–72.0)**	**64.3 (62.1–66.6)**	**65.8 (65.2–66.5)**	**64.4 (63.4–65.4)**	**63.7 (62.1–65.4)**
Median (range)	68 (6–85)	68 (4–85)	63 (12–85)	68 (23–85)	73 (33–85)	65 (8–85)	67 (29–85)	66 (20–85)	64 (40–85)

**Insurance, % (95% CI)**									
Insured	90.1 (89.8–90.5)	**76.5 (74.5–78.5)**	**76.6 (75.1–78.0)**	**76.6 (73.1–80.1)**	**98.0 (96.4–99.6)**	**71.4 (62.9–79.9)**	**75.9 (73.2–78.5)**	**83.6 (80.2–87.0)**	**70.5 (62.6–78.3)**
Medicaid	7.5 (7.2–7.8)	**20.1 (18.3–22.0)**	**18.3 (17.0–19.6)**	**21.7 (18.3–25.1)**	**1.3 (0.0–2.6)**	**21.4 (13.7–29.1)**	**21.4 (18.8–23.9)**	**13.1 (10.0–16.2)**	**25.8 (18.2–33.3)**
Uninsured	1.8 (1.7–2.0)	**2.4 (1.7–3.1)**	**4.0 (3.3–4.6)**	1.2 (0.3–2.1)	**0.3 (0.0–1.0)**	6.2 (1.7–10.8)	2.7 (1.7–3.7)	2.4 (1.0–3.8)	0.8 (0.0–2.3)
Unknown	0.5 (0.5–0.6)	1.0 (0.5–1.4)	**1.2 (0.8–1.5)**	0.5 (0.0–1.0)	0.3 (0.0–1.0)	0.9 (0.0–2.7)	**0.1 (0.0–0.3)**	0.9 (0.0–1.7)	3.0 (0.1–6.0)

**Income, % (95% CI)**									
<50K	31.6 (31.1–32.2)	**18.3 (16.5–20.1)**	**40.5 (38.8–42.1)**	**1.8 (0.7–2.8)**	**2.6 (0.8–4.5)**	**6.5 (2.3–12.0)**	**4.3 (3.0–5.5)**	**5.3 (3.2–7.3)**	**24.2 (16.8–31.6)**
50K–74K	51.3 (50.8–51.9)	**61.8 (59.6–64.1)**	50.7 (49.0–52.4)	54.8 (50.6–58.9)	**87.4 (83.7–91.2)**	46.4 (37.0–55.8)	**66.0 (63.0–68.9)**	**70.0 (65.8–74.2)**	**70.5 (62.6–78.3)**
>75K	17.0 (16.6–17.5)	**19.8 (18.0–21.7)**	**8.8 (7.8–9.7)**	**43.5 (39.4–47.6)**	**9.9 (6.5–13.3)**	**46.4 (37.0–55.8)**	**29.8 (26.9–32.6)**	**24.7 (20.8–28.7)**	**5.3 (1.4–9.2)**

**Sex, % (95% Cl)**									
Female	50.0 (49.4–50.6)	**54.5 (52.2–56.8)**	51.4 (49.7–52.6)	47.7 (43.6–51.8)	**62.3 (56.8–67.7)**	42.0 (32.7–51.2)	49 (45.9–52.0)	54.3 (49.7–58.9)	47.7 (39.1–56.3)
Male	50.0 (49.4–50.6)	**45.5 (43.2–47.8)**	48.6 (46.9–50.3)	52.3 (48.2–56.4)	**37.7 (32.2–43.2)**	58.0 (48.8–67.3)	51.0 (48.0–54.1)	45.7 (41.1–50.3)	52.3 (43.6–60.9)

**Marital Status**									
Married (including common law)	58.9 (58.3–59.5)	**54.2 (51.9–56.6)**	**37.2 (35.5–38.8)**	**74.6 (71.1–78.2)**	63.6 (58.1–69.0)	**73.2 (66.9–81.5)**	**71.1 (68.3–73.9)**	60.0 (56.4–64.5)	50.0 (41.4–58.6)
Single (never married)	9.5 (9.1–9.8)	**15.3 (13.7–17.0)**	**26.8 (25.3–28.3)**	**6.7 (4.6–8.8)**	**6.0 (3.3–8.6)**	8.9 (3.6–14.3)	7.6 (6.0–9.3)	10.1 (7.3–12.8)	12.1 (7.2–17.9)
Widowed	15.1 (14.7–15.6)	14.6 (12.9–16.2)	**12.9 (11.8–14.1)**	**9.2 (6.8–11.5)**	19.9 (15.3–24.4)	**5.4 (1.1–9.6)**	**12.1 (10.1–14.1)**	14.2 (11.0–17.4)	12.1 (7.2–17.9)
Other	16.5 (16.1–17.0)	15.9 (14.2–17.6)	**23.0 (21.6–24.5)**	**9.5 (7.1–11.9)**	**10.6 (7.1–14.1)**	12.5 (6.3–18.7)	**9.2 (7.4–11.0)**	15.8 (12.4–19.1)	**25.8 (18.2–33.3)**

**Stage, % (95% CI)**									
1	63.2 (62.6–63.7)	**59.2 (56.9–61.5)**	**58.3 (56.6–60.0)**	61.3 (57.2–65.3)	59.3 (53.7–64.8)	65.2 (56.2–74.1)	61.7 (58.7–64.7)	66.3 (62.0–70.7)	56.1 (47.5–64.6)
2	12.9 (12.5–13.3)	12.5 (10.9–14.0)	**14.5 (13.3–15.7)**	11.8 (9.1–14.5)	11.9 (8.2–15.6)	**7.1 (2.3–12.0)**	11.3 (9.3–13.2)	12.9 (9.8–16.0)	17.4 (10.9–24.0)
3	17.0 (16.6–17.5)	18.5 (16.7–20.4)	**19.0 (17.7–20.4)**	17.4 (14.3–20.6)	20.5 (15.9–25.1)	22.3 (14.5–30.2)	**20.2 (17.7–22.7)**	15.8 (12.4–19.1)	22.0 (14.8–29.1)
4	6.9 (6.6–7.2)	**9.8 (8.4–11.2)**	8.1 (7.1–9.1)	9.5 (7.1–11.9)	8.3 (5.2–11.4)	5.4 (1.1–9.6)	6.8 (5.3–8.4)	5.0 (3.0–7.0)	4.5 (0.9–8.1)

**Histology, % (95% CI)**									
Adenocarcinoma	60.2 (59.6–60.7)	**66.9 (64.8–69.1)**	**62.6 (61.0–64.3)**	**78.3 (74.9–81.7)**	**66.9 (61.5–72.2)**	**77.7 (69.8–85.5)**	**78.5 (76.0–81.1)**	**71.8 (67.6–75.9)**	**47.0 (38.3–55.6)**
Adenosquamous	2.9 (2.7–3.1)	3.4 (2.6–4.3)	2.9 (2.3–3.5)	2.8 (1.5–4.2)	3.0 (1.1–4.9)	**0.9 (0.0–2.7)**	2.2 (1.3–3.1)	1.8 (0.5–3.0)	3.8 (0.5–7.1)
Large cell	3.1 (2.9–3.3)	2.4 (1.7–3.1)	3.6 (3.0–4.3)	2.5 (1.2–3.7)	3.3 (1.3–5.3)	5.4 (1.1–9.6)	**1.2 (0.5–1.9)**	2.0 (0.7–3.2)	4.5 (0.9–8.1)
Non-small cell	3.7 (3.5–3.9)	(2.3–3.7)	**5.4 (4.6–6.1)**	2.6 (1.3–4.0)	2.3 (0.6–4.0)	2.7 (0.0–5.7)	**2.0 (1.1–2.8)**	**1.8 (0.5–3.0)**	8.3 (3.6–13.1)
Others	0.9 (0.8–1.1)	**1.7 (1.1–2.4)**	1.1 (0.7–1.4)	1.1 (0.2–1.9)	0.7 (0.0–1.6)	0.9 (0.0–2.7)	0.7 (0.2–1.2)	2.0 (0.7–3.2)	0.8 (0.0–2.3)
Squamous	29.3 (28.7–29.8)	**22.7 (20.8–24.7)**	**24.4 (22.9–25.9)**	**12.7 (9.9–15.4)**	**23.8 (19.0–28.7)**	**12.5 (6.3–18.7)**	**15.4 (13.2–17.7)**	**20.8 (17.1–24.5)**	35.6 (27.3–43.9)

**Surgical category, % (95% CI)**									
(Bi)Lobectomy	75.7 (75.2–76.2)	76.5 (74.5–78.5)	75.8 (74.3–77.2)	**86.1 (83.2–88.9)**	80.4 (76.0–85.0)	80.4 (72.9–87.8)	**81.4 (79.0–83.8)**	77.9 (74.1–81.7)	77.3 (70.0–84.5)
Pneumonectomy	5.7 (5.4–6.0)	5.5 (4.4–6.5)	5.4 (4.6–6.2)	**2.5 (1.2–3.7)**	3.6 (1.5–5.8)	3.6 (0.1–7.1)	**3.9 (2.7–5.0)**	4.6 (2.7–6.5)	9.1 (4.1–14.1)
Segmentectomy	3.1 (2.9–3.3)	3.2 (2.4–4.0)	2.9 (2.3–3.4)	2.3 (1.1–3.5)	3.6 (1.5–5.8)	**0.9 (0.0–2.7)**	2.7 (1.7–3.7)	**1.8 (0.5–3.0)**	2.3 (0.0–4.8)
Sub-lobar resection, NOS	0.6 (0.5–0.7)	0.9 (0.5–1.3)	1.0 (0.6–1.3)	0.4 (0.0–0.8)	0.7 (0.0–1.6)	0.9 (0.0–2.7)	0.5 (0.1–0.9)	1.1 (0.1–2.1)	NA
Wedge Resection	14.9 (14.5–15.4)	13.9 (12.3–15.6)	15.0 (13.8–16.2)	**8.8 (6.5–11.1)**	11.6 (8.0–15.2)	14.3 (7.7–20.9)	**11.6 (9.6–13.5)**	14.7 (11.4–17.9)	11.4 (5.9–16.8)

**Sub-lober > 2 cm, % (95% CI)**									
No	92.6 (92.3–92.9)	(91.5–93.8)	(91.3–93.1)	**95.0 (93.3–96.7)**	(89.4–95.2)	(87.0–96.8)	(91.3–94.4)	(92.7–96.7)	(91.1–98.3)
Yes	7.4 (7.1–7.7)	7.4 (6.2–8.5)	7.8 (6.9–8.7)	**5.0 (3.3–6.7)**	7.7 (4.8–10.6)	8.1 (3.2–13.0)	7.1 (5.6–8.7)	5.3 (3.3–7.3)	5.3 (1.7–8.9)

**Number of nodes examined**									
Mean (95% CI)	9.0 (8.9–9.1)	**8.5 (8.2–8.9)**	**8.2 (8.0–8.5)**	9.0 (8.3–9.7)	9.0 (8.1–9.8)	9.9 (8.0–11.8)	**8.3 (7.8–8.7)**	8.6 (7.9–9.3)	8.3 (6.7–9.9)
Median (range)	7 (0–90)	7 (0–87)	6 (0–90)	7 (0–90)	7 (0–44)	6.5 (0–68)	6 (0–60)	7 (0–67)	6 (0–54)

**Number of nodes positive**									
Mean (95% CI)	0.6 (0.6–0.7)	0.7 (0.6–0.7)	0.6 (0.6–0.7)	0.7 (0.5–0.9)	0.8 (0.6–1.1)	0.8 (0.4–1.2)	0.7 (0.6–0.9)	0.8 (0.6–0.9)	0.8 (0.5–1.2)
Median (range)	0 (0–61.0)	0 (0–26.0)	0 (0–29.0)	0 (0–28.0)	0 (0–21.0)	0 (0–13.0)	0 (0–16.0)	0 (0–24.0)	0 (0–17.0)

**Node positivity, % (95% CI)**									
No	78.2 (77.7–78.6)	77.5 (75.6–79.5)	76.7 (75.3–78.1)	74.5 (70.9–78.1)	73.8 (68.9–78.8)	75.9 (67.8–83.9)	**(71.3–76.7)**	76.8 (72.9–80.7)	74.2 (66.7–81.8)
Yes	21.8 (21.4–22.3)	22.5 (20.5–24.4)	23.3 (21.9–24.7)	25.5 (21.9–29.1)	26.2 (21.2–31.1)	24.1 (16.1–32.2)	**26.0 (23.3–28.7)**	23.2 (19.3–27.1)	25.8 (18.2–33.3)

**Node density**									
Mean (95% CI) (0.06–0.07)	0.07 (0.06–0.07)	0.08 (0.07–0.09)	0.08 (0.07–0.08)	0.09 (0.07–0.11)	0.08 (0.06–0.10)	0.08 (0.04–0.11)	**0.10 (0.08–0.11)**	0.08 (0.07–0.10)	0.08 (0.05–0.10)
Median (range)	0 (0–1.0)	0 (0–1.0)	0 (0–1.0)	0 (0–1.0)	0 (0–1.0)	0 (0–1.0)	0 (0–1.0)	0 (0–1.0)	0 (0–0.9)

**30-day survival, % (95% CI)**									
No	1.1 (1.0–1.2)	**1.8 (1.2–2.4)**	1.1 (0.7–1.4)	1.1 (0.2–1.9)	**0.3 (0.0–1.0)**	3.3 (0.1–6.4)	1.3 (0.6–2.0)	**0.2 (0.0–0.7)**	3.1 (0.1–6.0)
Yes	98.9 (98.8–99.0)	**98.2 (97.6–98.8)**	98.9 (98.6–99.3)	98.9 (98.1–99.8)	**99.7 (99.0–100.0)**	100.0 (93.6–99.9)	98.7 (98.0–99.4)	**99.8 (99.3–100.0)**	96.9 (94.0–100.0)

**90-day survival, % (95% CI)**									
No	4.1 (3.9–4.4)	5.2 (4.1–6.3)	4.0 (3.3–4.7)	2.8 (1.4–4.2)	2.4 (0.6–4.2)	1.9 (0.0–4.5)	**2.7 (1.7–3.7)**	**1.9 (0.6–3.2)**	6.9 (2.5–11.3)

**Cancer death, % (95% CI)**									
No	76.6 (76.1–77.1)	77.7 (75.7–79.6)	76.1 (74.7– 77.6)	**82.7 (79.6–85.9)**	75.8 (71.0–80.7)	**85.7 (79.1–92.3)**	**81.1 (78.7–83.5)**	**84.9 (81.6–88.2)**	81.1 (74.3–87.8)
Yes	23.4 (22.9–23.9)	22.3 (20.4–24.3)	23.8 (22.4–25.3)	**17.3 (14.1–20.4)**	24.2 (19.3–29.0)	14.3 (7.7–20.3)	**18.9 (16.5–21.3)**	**15.1 (11.8–18.4)**	18.9 (12.2–25.7)

**Other cause death, % (95% CI)**									
No	91.9 (91.6–92.2)	**93.5 (92.4–94.7)**	92.4 (91.5–93.3)	**94.9 (93.1–96.7)**	91.4 (88.2–94.6)	94.6 (90.4–98.9)	**94.0 (92.5–95.4)**	**95.0 (93.0–97.0)**	90.9 (85.9–95.9)
Yes	8.1 (7.8–8.4)	**6.5 (5.3–7.6)**	7.6 (6.7–8.5)	**5.1 (3.3–6.9)**	8.6 (5.4–11.8)	5.4 (1.1–9.6)	**6.0 (4.6–7.5)**	**5.0 (3.0–7.0)**	9.1 (4.1–14.1)

*95% confident intervals are given in parentheses. W is used as reference population. All characteristics differing from the W are in bold-print and have brown colored backgrounds. Otherwise, green and blue depict individual rows are different colors for ease of visualization*.

### OS in the Total Surgical Population

**Figure 2:**
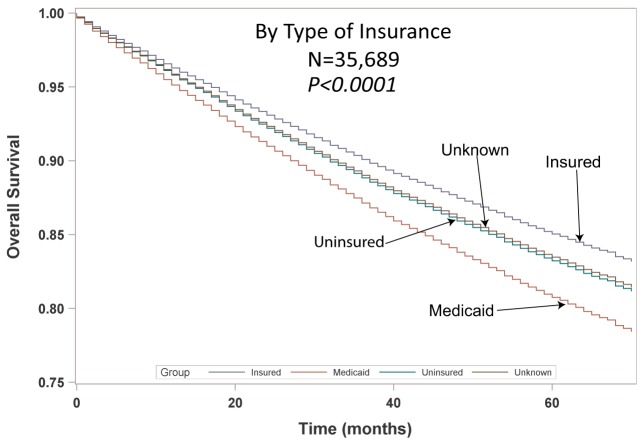
Multivariable adjusted overall survival by insurance type in TS population.

Multivariable analysis (MVA) for OS for TS population can be seen in Table 3. Age (*p* < 0.0001, HR = 1.029) and male sex (*p* < 0.0001, HR = 1.453) were significantly associated with OS. OS was significantly better than Whites (HR = 0.693–0.843) in all groups except for AI/ANs, Japanese, Blacks, and Hispanics who had a similar OS. MVA-adjusted OS by ethic group can be seen in Figure 1B. As compared to Connecticut, worse survival was noted in California, Greater Georgia, Iowa, Kentucky, Louisiana, and Utah. OS was not income dependent. Insured patients had a better OS than those on Medicaid (*p* < 0.0001, HR = 1.286). Married patients had a better OS than divorced (*p* < 0.0001, HR = 1.191), widowed (*p* < 0.0001, HR = 1.229), and single patients (*p* < 0.0001, HR = 1.1215). As compared to Stage I, Stages II–IV were associated with a worse OS with a progressively increasing HR (all *p* < 0.0001, HR = 1.702–3.273). As compared to patients with adenocarcinoma, all histologies were associated with a worse OS (*p* < 0.0001 to <0.0008, HR = 1.119–1.564). Using well-differentiated tumors as a reference, all other tumor grades were associated with a worse OS (all *p* < 0.0001, HR = 1.665–3.273). Segmentectomies and (bi)lobectomies were associated with a better OS than pneumonectomies, *p* = 0.0011, HR = 0.80; *p* < 0.0001, HR = 0.72, respectively. Patients who received radiation (*p* < 0.0001, HR = 1.162) experienced worse OS. Number of nodes examined was associated with better OS (*p* < 0.0001, HR = 0.988), but number of nodes positive (*p* < 0.0001, HR = 1.04) and lymph node density (*p* < 0.0001, HR = 1.429) were associated with worse OS. Compared to year 2007, those patients diagnosed in 2010–2012 had significantly better OS with progressively decreasing hazard ratios. OS by insurance status can be seen in Figure 2.

**Table 3:** Multivariate analysis of overall survival in the TS population.

All surgical (*N* **=** 35,689)	*p*-Value	Hazard ratio
Age—years	<0.0001	1.029

**Sex**		
Female	–	1
Male	<0.0001	1.453

**Race**		
White Hispanic	0.49	0.968
White non-Hispanic	–	1
Black	0.46	1.026
Chinese	<0.0001	0.693
Japanese	0.06	1.027
South Asian	0.01	0.843
Other Asian	0.01	0.843
Other Race	0.02	0.772
American Indian/Alaskan Native	0.74	1.065

**Surveillance Epidemiology and End Reporting Registry**		
Alaska Natives	0.72	0.873
Atlanta	0.40	1.062
California excl SF/SJM/LA	0.001	1.167
Connecticut	–	1
Detroit	0.95	0.996
Greater Georgia	0.0005	1.217
Hawaii	0.33	1.102
Iowa	0.01	1.176
Kentucky	0.0001	1.249
Los Angeles	0.06	1.111
Louisiana	0.004	1.198
New Jersey	0.12	1.081
New Mexico	0.22	1.127
Rural Georgia	0.82	1.049
San Francisco-Oakland	0.12	1.107
San Jose-Monterey	0.49	1.059
Seattle	0.07	1.126
Utah	0.008	1.269

**Income**		
<$50,000	0.05	1.06
$50,000–75,000	–	1
>75,000	0.24	0.963

**Marital status**		
Divorced	<0.0001	1.191
Married	–	1
Separated	0.18	1.144
Single	<0.0001	1.215
Unknown	0.05	1.118
Domestic partner	0.67	0.783
Widowed	<0.0001	1.229

**Stage**	
I	–	1
II	<0.0001	1.702
III	<0.0001	1.867
IV	<0.0001	3.273

**Insurance**		
Insured	–	1
Medicaid	<0.0001	1.286
Uninsured	0.08	1.135
Unknown	0.33	1.286

**Lateral location**		
Bronchus, Left	0.92	1.014
Bronchus, right	0.01	1.42
Bronchus, unknown	0.33	0.613
Left lower	0.08	1.056
Left upper	0.10	1
Left NOS	0.04	1.211
Left overlapping	0.15	0.801
Lung, NOS	<0.0001	2.061
Right lower	<0.0001	1.23
Right middle	0.75	1.015
Right upper	–	1
Right NOS	0.45	1.062
Right overlapping	<0.0001	1.371

**Histology—no. (%)**		
Adenocarcinoma	–	1
Adenosquamous	0.0008	1.196
Large cell	<0.0001	1.348
Non-small cell	0.0003	1.174
Other	<0.0001	1.564
Squamous	<0.0001	1.159

**Grade**		
Moderately, II	<0.0001	1.702
Poorly, III	<0.0001	1.867
Undifferentiated, IV	<0.0001	3.273
Unknown	<0.0001	1.665
Well, I	–	1

**Surgical procedure**		
(Bi)Lobectomy	<0.0001	0.721
No surgery		
Pneumonectomy	–	1
Segmentectomy	0.0011	0.800
Sub-lobar resection, NOS	0.13	1.172
Wedge	0.63	0.978

Radiation post-operative	<0.0001	1.162
Number of nodes examined	<0.0001	0.988
Number of nodes positive	<0.0001	1.04
Node density	<0.0001	1.429

**Year of diagnosis—no. (%)**		
2007	–	1
2008	0.95	1.002
2009	0.28	0.969
2010	0.02	0.927
2011	0.0018	0.888
2012	<0.0001	0.787

### OS in the ESR Population

Multivariable analysis for OS for ESR population can be seen in Table 4. Age (*p* < 0.0001, HR = 1.034), and male sex (*p* < 0.0001, HR = 1.506) were significantly associated with OS. OS was significantly better than Whites in the Other Race (*p* = 0.0051, HR = 0.555) and Other Asian groups (*p* = 0.012, HR = 0.736), but it was similar in all other ethnic groups. As compared to Connecticut, worse survival was noted in California, Greater Georgia, Kentucky, Louisiana, and Utah. OS was not income dependent. Insured patients had a better OS than those on Medicaid (*p* < 0.0001, HR = 1.385). Married patients had a better OS than divorced (*p* < 0.0001, HR = 1.301), widowed (*p* < 0.0001, HR = 1.292), and single patients (*p* = 0.0015, HR = 1.121). Increasing tumor size (*p* < 0.0001, HR = 1.016) and T2 vs T1 (*p* < 0.0129, HR = 1.107) had a worse OS. Only the right lower lobe location was associated with survival (*p* < 0.0089, HR = 1.132). In comparison to patients with adenocarcinoma, large cell carcinoma, NSCLC-NOS, and squamous cell carcinoma were associated with a worse OS (*p* < 0.0011 to <0.0001, HR = 1.15–1.381). Using well-differentiated tumors as a reference, all other tumor grades were associated with a worse OS (HR = 1.572–1.846). Segmentectomies (*p* < 0.0090, HR = 1.235), pneumonectomies (*p* < 0.0001, HR = 1.782), and wedge resections (*p* < 0.0001, HR = 1.301) were associated with a worse OS than (bi)lobectomies. Patients who received radiation (*p* < 0.0001, HR = 1.36) experienced worse OS. Number of nodes examined was associated with better OS (*p* < 0.0001, HR = 0.984). Compared to year 2007, those patients diagnosed in 2010 and 2012 had significantly better OS.

**Table 4:** Multivariate analysis for overall survival in early-stage resectable population.

Early-stage resectable (*N* **=** 17,931)	*p*-Value	Hazard ratio
Age—years	<0.0001	1.034

**Sex**		
Female	–	1
Male	<0.0001	1.506

**Race**		
White Hispanic	0.08	0.856
White non-Hispanic	–	1
Black	0.80	0.984
Chinese	0.15	0.787
Japanese	0.19	0.757
South Asian	0.35	0.702
Other Asian	0.012	0.736
Other Race	0.0051	0.555
American Indian/Alaskan Native	0.69	0.873

**Surveillance Epidemiology and End Reporting Registry**		
Alaska Natives	0.63	0.598
Atlanta	0.47	1.098
California excl SF/SJM/LA	0.05	1.173
Connecticut	–	1
Detroit	0.57	1.06
Greater Georgia	0.004	1.312
Hawaii	0.11	1.32
Iowa	0.70	1.043
Kentucky	0.0099	1.286
Los Angeles	0.77	1.028
Louisiana	0.03	1.258
New Jersey	0.22	1.108
New Mexico	0.38	1.158
Rural Georgia	0.13	0.503
San Francisco-Oakland	0.61	1.06
San Jose-Monterey	0.95	0.99
Seattle	0.18	1.154
Utah	0.05	1.397

**Income**		
<$50,000	0.71	1.019
$50,000–74,000	–	1
≥75,000	0.19	0.93

**Marital status**		
Divorced	<0.0001	1.301
Married	–	1
Separated	0.23	1.239
Single	0.0015	1.211
Unknown	0.54	1.062
Domestic partner	0.84	1.221
Widowed	<0.0001	1.292

Tumor size	<0.0001	1.016

**Tumor stage**		
T1	–	1
T2	0.01	1.107

**Insurance**		
Insured	–	1
Medicaid	<0.0001	1.385
Uninsured	0.69	1.065
Unknown	0.67	0.887

**Lateral location**		
Bronchus, left	0.29	0.468
Bronchus, right	0.87	0.891
Bronchus, unknown	0.89	0.872
Left lower	0.36	0.952
Left upper	0.92	1.004
Left NOS	0.14	1.454
Left overlapping	0.90	1.055
Right lower	0.0089	1.132
Right middle	0.09	0.869
Right upper	–	1
Right NOS	0.84	0.946
Right overlapping	0.75	0.926

**Histology—no. (%)**		
Adenocarcinoma	–	1
Adenosquamous	0.16	1.15
Large cell	0.0011	1.381
Non-small cell	0.001	1.317
Other	0.78	0.942
Squamous	<0.0001	1.236

**Grade**		
Moderately, II	<0.0001	1.621
Poorly, III	<0.0001	1.846
Undifferentiated, IV	0.0019	1.572
Unknown	<0.0001	1.707
Well, I	–	1

**Surgical procedure**		
(Bi)Lobectomy	–	1
Pneumonectomy	<0.0001	1.782
Segmentectomy	0.009	1.235
Sub-lobar resection, NOS	0.10	1.442
Wedge	<0.0001	1.301

Radiation post-operative	<0.0001	1.36

Number of nodes examined	<0.0001	0.984

**Year of diagnosis**		
2007	–	1
2008	0.03	0.904
2009	0.13	0.929
2010	0.0016	0.832
2011	0.44	0.949
2012	0.0004	0.661

### LCSS in the ESR Population

**Figure 3:**
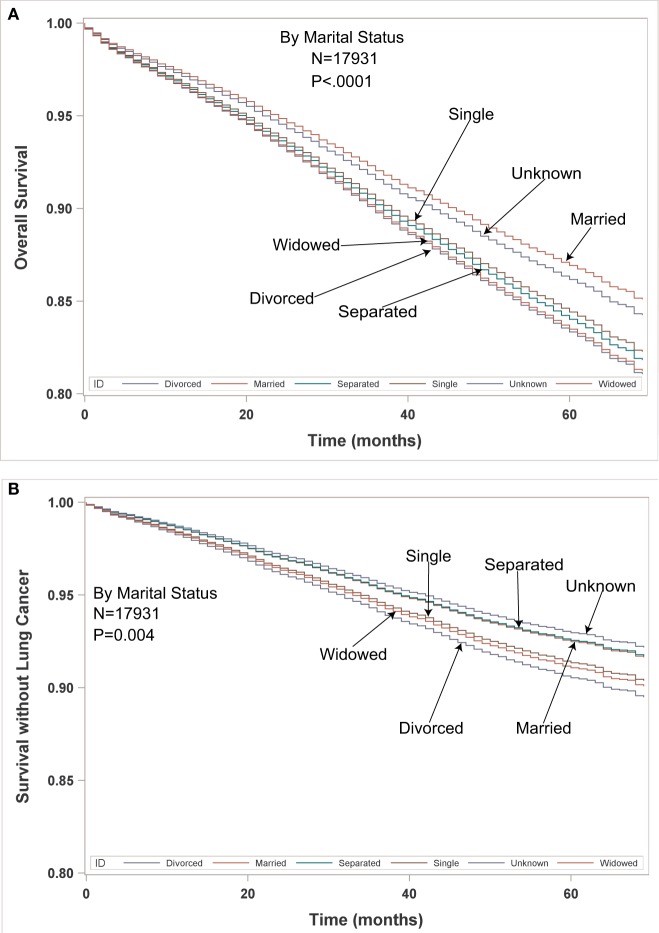
**(A,B)** Multivariable adjusted overall survival and lung cancer-specific survival in the early-stage resectable population by marital status.

Multivariate analysis for LCSS for ESR population can be seen in Table 5. Age (*p* < 0.0001, HR = 1.023) and male sex (*p* < 0.0001, HR = 1.393) were significantly associated with LCSS. LCSS was not significantly associated with race or income. As compared to Connecticut, worse LCSS was noted in Greater Georgia, Kentucky, and Louisiana. Insured patients had a better LCSS than those on Medicaid (*p* < 0.0001, HR = 1.445). Married patients had a better LCSS than divorced (*p* < 0.0004, HR = 1.301) and widowed (*p* < 0.0036, HR = 1.200). Increasing tumor size (*p* < 0.0001, HR = 1.020) and T2 vs T1 (*p* = 0.0003, HR = 1.213) were associated with a worse LCCS. Only the right middle lobe location was associated with LCSS (*p* < 0.0469, HR = 0.803). As compared to patients with adenocarcinoma, NSCLC-NOS (*p* < 0.002, HR = 1.382) and large cell carcinoma (*p* = 0.0003, HR = 1.543) were correlated with a worse LCSS. Using well-differentiated tumors as a reference, all other tumor grades were associated with a worse LCSS (HR = 1.693–2.171). Segmentectomies (*p* < 0.0065, HR = 1.329), pneumonectomies (*p* = 0.0027, HR = 1.781), and wedge resections (*p* < 0.0001, HR = 1.353) were associated with a worse LCSS than (bi)lobectomies. Patients who received radiation (*p* < 0.0001, HR = 1.556) experienced worse LCSS. Number of nodes examined was associated with better LCSS (*p* < 0.0001, HR = 0.978). Compared to year 2007, those patients diagnosed in all other years, except for 2011 had a significantly better LCSS. OS and LCSS by marital status can be seen in Figures 3A,B.

**Table 5:** Multivariate analysis for lung cancer-specific survival in early-stage resectable population.

Early-stage resectable (*N* **=** 17,931)	*p*-Value	Hazard ratio
Age—years	<0.0001	1.023

**Sex**		
Female		1
Male	<0.0001	1.393

**Race**		
White Hispanic	0.26	0.877
White non-Hispanic	–	1
Black	0.54	0.949
Chinese	0.41	0.839
Japanese	0.07	0.534
South Asian	0.76	0.872
Other Asian	0.06	0.745
Other Race	0.10	0.655
American Indian/Alaskan Native	0.14	0.348

**Surveillance Epidemiology and End Reporting Registry**		
Alaska Natives	0.44	2.575
Atlanta	0.82	0.96
California excl SF/SJM/LA	0.17	1.183
Connecticut	–	1
Detroit	0.36	1.13
Greater Georgia	0.02	1.344
Hawaii	0.60	1.134
Iowa	0.16	1.218
Kentucky	0.0025	1.473
Los Angeles	0.41	1.112
Louisiana	0.0065	1.457
New Jersey	0.55	1.07
New Mexico	0.05	1.497
Rural Georgia	0.36	0.585
San Francisco-Oakland	0.92	0.985
San Jose-Monterey	0.65	0.909
Seattle	0.76	1.046
Utah	0.31	1.278

**Income**		
<$50,000	0.17	0.912
$50,000–74,000	–	1
≥75,000	0.62	0.965

**Marital status**		
Divorced	0.0004	1.272
Married	–	1
Separated	0.96	0.988
Single	0.06	1.16
Unknown	0.61	0.935
Domestic partner	0.97	0
Widowed	0.0036	1.2

Tumor size	<0.0001	1.02

Tumor stage		
T1	–	1
T2	0.0003	1.213

**Insurance**		
Insured		1
Medicaid	<0.0001	1.445
Uninsured	0.89	1.029
Unknown	0.84	0.932

**Lateral location**		
Bronchus, left	0.92	0
Bronchus, right	0.63	1.41
Bronchus, unknown	0.97	0
Left lower	0.68	0.971
Left upper	0.91	0.994
Left NOS	0.42	1.334
Left overlapping	0.77	1.16
Right lower	0.09	1.111
Right middle	0.05	0.803
Right upper	–	1
Right NOS	0.86	0.941
Right overlapping	0.91	0.966

**Histology—no. (%)**		
Adenocarcinoma	–	1
Adenosquamous	0.43	1.106
Large cell	0.0003	1.543
Non-small cell	0.002	1.382
Other	0.87	0.957
Squamous	0.06	1.104

**Grade**		
Moderately, II	<0.0001	1.81
Poorly, III	<0.0001	2.171
Undifferentiated, IV	0.005	1.693
Unknown	<0.0001	2.013
Well, I	–	1

**Surgical procedure**		
(Bi)Lobectomy	–	1
Pneumonectomy	0.003	1.781
Segmentectomy	0.007	1.329
Sub-lobar resection, NOS	0.08	1.61
Wedge	<0.0001	1.353

Radiation post-operative	<0.0001	1.556

Number of nodes examined	<0.0001	0.978

**Year of diagnosis—no. (%)**		
2007	–	1
2008	0.02	0.875
2009	0.02	0.857
2010	0.001	0.779
2011	0.06	0.842
2012	0.003	0.612

### 90-Day Mortality Analysis

Multivariate analysis for 90-day OS for TS population can be seen in Table 6. Age (*p* < 0.0001, HR = 1.045) and male sex (*p* < 0.0001, HR = 1.547) were significantly associated with 90-day OS. 90-day mortality was the same in all ethnic groups. Higher median income (>$75,000) was associated with a better survival. As compared to Connecticut, worse survival was noted in Louisiana and Utah. Insured patients had a better 90-day OS than those on Medicaid (*p* = 0.0005, HR = 1.359) and those with unknown insurance (*p* = 0.0003, HR = 2.774). Married patients had a better OS than single (*p* = 0.0188, HR = 1.239) and unmarried/domestic partner patients (*p* = 0.0310, HR = 3.523). Right bronchus (*p* = 0.0001, HR = 2.652), bronchus unknown (*p* = 0.0012, HR = 6.926), and right lower lobe (*p* < 0.0001, HR = 1.386) were associated with worse 90-day mortality than the right upper lobe location. As compared to Stage I, Stages II–IV were associated with a worse OS with a progressively increasing HRs (all *p* < 0.0001, HR = 1.607–4.381). As compared to patients with adenocarcinoma, NSCLC-NOS (*p* < 0.0034, HR = 1.460), other (*p* < 0.0001, HR = 2.334), and squamous cell carcinoma (*p* < 0.0001, HR = 1.436) had a higher risk of 90-day mortality. Using well-differentiated tumors as a reference, 90-day mortality was higher in patients having poorly differentiated, undifferentiated, and unknown differentiated tumors. Pneumonectomies were associated with a significantly higher 90-day mortality than all other resection types (*p* = 0.0281 to <0.0001, HR = 0.418–0.775), except for sub-lobar, NOS which had a higher mortality (*p* = 0.0012, HR = 1.885). Patients who received radiation experienced a significantly lower 90-day mortality (*p* < 0.0001, HR = 0.217). Number of nodes examined was associated with better OS (*p* = 0.0001, HR = 0.984), but number of nodes positive and lymph node density were associated with worse OS. Similar 90-day mortality was noted to 2007 for years 2008–2012.

**Table 6:** Multivariate analysis for 90-day overall survival in TP.

All surgical (*N* **=** 35,689)	*p*-Value	Hazard ratio
Age—years	<0.0001	1.045

**Sex**		
Female	–	1
Male	<0.0001	1.547

**Race**		
White Hispanic	0.09	1.219
White non-Hispanic	–	1
Black	0.96	1.005
Chinese	0.58	0.861
Japanese	0.11	0.532
South Asian	0.58	0.672
Other Asian	0.21	0.772
Other Race	0.11	0.563
American Indian/Alaskan Native	0.11	1.861

**Surveillance Epidemiology and End Reporting Registry**		
Alaska Natives	0.80	0.813
Atlanta	0.34	1.212
California excl SF/SJM/LA	0.51	1.098
Connecticut	–	1
Detroit	0.41	0.858
Greater Georgia	0.10	1.301
Hawaii	0.91	1.036
Iowa	0.91	1.022
Kentucky	0.09	1.317
Los Angeles	0.13	1.271
Louisiana	0.04	1.428
New Jersey	0.39	1.134
New Mexico	0.25	1.332
Rural Georgia	0.16	1.854
San Francisco-Oakland	0.96	0.99
San Jose-Monterey	0.79	1.068
Seattle	0.10	1.347
Utah	0.01	1.735

**Income**		
<$50,000	0.07	1.16
$50,000–75,000	–	1
>75,000	0.01	0.782

**Tumor stage**		
I	–	1
II	<0.0001	1.607
III	<0.0001	2.238
IV	<0.0001	4.381

**Marital status**		
Divorced	0.78	0.974
Married	–	1
Separated	0.88	0.954
Single (never married)	0.02	1.239
Unknown	0.52	1.1
Unmarried or domestic partner	0.03	3.523
Widowed	0.13	1.127

**Insurance**		
Insured	–	1
Medicaid	0.0005	1.359
Uninsured	0.24	1.279
Unknown	0.0003	2.774

**Lateral location**		
Bronchus, left	0.51	0.786
Bronchus, right	0.0001	2.652
Bronchus, unknown	0.0012	6.926
Left lower	0.37	0.917
Left upper	0.43	1.063
Left NOS	0.01	1.628
Left overlapping	0.34	1.369
Lung, NOS	0.0004	2.37
Right lower	<0.0001	1.386
Right middle	0.80	0.965
Right upper	–	1
Right NOS	0.004	1.587
Right overlapping	<0.0001	2.725

**Histology—no. (%)**		
Adenocarcinoma	–	1
Adenosquamous	0.79	1.044
Large cell	0.42	1.139
Non-small cell	0.003	1.46
Other	<0.0001	2.334
Squamous	<0.0001	1.436

**Grade**		
Moderately, II	0.23	1.134
Poorly, III	0.003	1.378
Undifferentiated, IV	0.005	1.745
Unknown	0.0004	1.584
Well, I	–	1

**Surgical procedure**		
Sub-lobar resection, NOS	0.001	1.885
(Bi)lobectomy	<0.0001	0.475
Pneumonectomy	–	1
Segmentectomy	<0.0001	0.418
Wedge	0.03	0.775

Radiation post-operative	<0.0001	0.217

Number of nodes examined	0.0001	0.984

Number of nodes positive	0.51	0.986

Node density	0.08	1.352

**Year of diagnosis—no. (%)**		
2007	–	1
2008	0.35	1.087
2009	0.41	1.077
2010	0.82	1.021
2011	0.96	0.996
2012	0.53	1.065

### Characteristics Associated With Nodal Positivity

In Table 7, a multivariate analysis was performed for the risk of having nodal positivity in patients undergoing a definitive surgical procedure with a T1–T2 tumor <2 cm and at least one lymph node examined. The results were adjusted for type of surgical resection. Age (*p* < 0.0001, HR = 1.036) and male sex (*p* < 0.0001, HR = 1.386) were significantly associated with positive nodes. Positive nodes were not associated with any ethnic or income group. As compared to Connecticut, a greater risk of positive nodes was found in Greater Georgia, Hawaii, and Utah. T2 tumor had a higher risk of positive nodes than T1 tumors (*p* = 0.0004, HR = 1.289). Patients without a married partner (*p* < 0.0033, HR = 1.376) or without insurance (*p* < 0.0003, HR = 1.376) were more likely to have positive nodes. Right lower lobe location (*p* < 0.0353, HR = 1.185) was associated with a higher likelihood of positive nodes than the right upper lobe location. As compared to patients with adenocarcinoma, adenosquamous cell (*p* < 0.0316, HR = 1.416), large cell (*p* < 0.0252, HR = 1.426), and squamous cell carcinomas (*p* = 0.0437, HR = 1.149) had a higher risk of having positive nodes. Using well-differentiated tumors as a reference, nodal positivity was higher in patients having poorly differentiated (*p* < 0.0001, HR = 2.157), moderately differentiated (*p* < 0.0001, HR = 1.784), and unknown differentiated tumors (*p* < 0.0001, HR = 1.802). Number of nodes examined was not associated with nodal positivity. Nodal positivity was less likely in years 2010–2012 (*p* = 0.0427–0.0027), with a progressively decreased HR (0.821–0.0027).

**Table 7:** Multivariate analysis for node positivity by various factors for T1–T2 tumors <2 cm with at least one node removed, adjusted for type of surgical resection.

All surgical patients with T1 or T2 tumors **<**2 cm (*N* **=** 7,580)	*p*-Value	Hazard ratio
Age—years	<0.0001	1.036

**Sex**		
Female	–	1
Male	<0.0001	1.386

**Race**		
White Hispanic	0.99	0.998
White non-Hispanic	–	1
Black	0.89	0.986
Chinese	0.06	0.488
Japanese	0.32	0.699
South Asian	0.70	0.675
Other Asian	0.31	0.808
Other Race	0.38	0.754
American Indian/Alaskan Native	0.09	1.219

**Surveillance Epidemiology and End Reporting Registry**		
Alaska Natives	0.97	1
Atlanta	0.38	1.19
California excl SF/SJM/LA	0.36	1.13
Connecticut	–	1
Detroit	0.59	0.912
Greater Georgia	0.02	1.415
Hawaii	0.05	1.83
Iowa	0.95	0.988
Kentucky	0.41	1.144
Los Angeles	0.77	0.953
Louisiana	0.49	1.136
New Jersey	0.93	1.012
New Mexico	0.43	1.256
Rural Georgia	0.90	1.099
San Francisco-Oakland	0.97	1.008
San Jose-Monterey	0.54	0.837
Seattle	0.11	1.323
Utah	0.04	1.705

**Income**		
<$50,000	0.10	1.151
$50,000–75,000	–	1
>75,000	0.39	0.922

Tumor size		1.008

**Tumor stage**		
T2 vs T1	0.0004	1.289

**Marital status**		
Other	0.003	1.191
Married	–	1

**Insurance**		
Insured	–	1
Other	0.0003	1.376

**Lateral location**		
Bronchus, left	0.95	1.047
Bronchus, right	0.95	1
Left lower	0.90	0.989
Left upper	0.62	0.965
Left NOS	0.83	1.088
Left overlapping	0.95	1.047
Lung, NOS	0.99	1
Right lower	0.04	1.185
Right middle	0.08	0.782
Right upper	–	1
Right NOS	0.30	0.653
Right overlapping	0.87	1.087

**Histology—no. (%)**		
Adenocarcinoma	–	1
Adenosquamous	0.03	1.419
Large cell	0.03	1.426
Non-small cell	0.46	1.113
Other	0.11	0.199
Squamous	0.04	1.149

**Grade**		
Moderately, II	<0.0001	1.784
Poorly, III	<0.0001	2.157
Undifferentiated, IV	0.89	1.047
Unknown	<0.0001	1.802
Well, I	–	1

Number of nodes examined	0.28	0.995

**Year of diagnosis—no. (%)**		
2007	–	1
2008	0.70	0.971
2009	0.71	0.969
2010	0.04	0.821
2011	0.003	0.679
2012	0.003	0.519

## Discussion

The purpose of our investigation was to assess difference in outcomes (OS and 30/90 day mortality), presentation, and treatment in nine different ethnic groups who underwent surgical resection of NSCLC. As compared to Whites, the unadjusted OS and LCSS was significantly greater in the Chinese, South Asian, Other Asian, and the Other Race groups. After multivariable adjustment, OS was significantly better than Whites in all groups except for AI/ANs, Japanese, Blacks, and Hispanics who had a similar OS. Despite presenting with higher stage tumors, lower median incomes, lower rates of insurance, less nodes examined, less grade 1 tumors, and lower marriage rates, the OS and LCSS of the Black group were not significantly different than that of the Whites. In comparison to the White group, Hispanics had a similar LCSS, but had an improved OS despite having a higher unadjusted 30-day mortality. Although Hispanics presented with a lower percentage of Stage I patients, lower marriage rates, less nodes examined, and lower rates of insurance, they presented with many better prognostic features compared to the Whites including higher income, lower tumor grades, younger age, higher percentage of female patients, and a higher percentage of adenocarcinomas. The Chinese and Other Asian groups were more likely to receive a (bi)lobectomy than the Whites, but the other ethnic groups largely did not differ in the type of surgical procedure. The reason for the higher 30-day mortality (unadjusted) in the Hispanic population is currently unknown, but the all other populations had a similar or better (Japanese or Other Race) 30-day survival to the White population. Although the unadjusted 90-day mortality was lower in the Other Asian and Other Race populations, there was no difference between the other ethnic groups and the Whites. However, the MVA demonstrated that there was no significant difference between the ethnic groups as compared to Whites. It should be noted that we included stage IV patients in this analysis of patients undergoing a definitive surgical procedure because a satellite nodule in a different lobe of the ipsilateral lung was classified by the AJCC staging as metastatic until 2010 when the new AJCC seventh edition classified this situation as T4 (11). The percentage of each ethnic group undergoing a definitive surgical procedure for Stage IV disease varied from 4.5 to 9.8%. Only the Hispanic group had significantly different percentage of Stage IV patients than the White patients (9.8% of Hispanics vs 6.9% of Whites). Two thousand five hundred sixty three patients with Stage IV tumors underwent a definitive surgical procedure. One thousand six hundred twenty-seven patients were classified as having tumors nodules in different ipsilateral lobes during the years 2007–2009. One thousand one hundred twenty-nine underwent a sub-lobar resection (966 wedge, 92 segmentectomy, and 71 sub-lobar, NOS). Although some patients may have undergone a diagnostic wedge procedure, we assume that most of the remaining patients who did not have tumor nodules in different ipsilateral lobes (*N* = 936) may have been found to have metastatic disease shortly after their surgical procedure. However, the performance of staging investigations and their timing in relation to surgical procedures is not available in SEER. Nevertheless, after removing the patients who would now be re-classified as having Stage III NSCLC, the numbers were too small for further characterization of these patients by ethnicity.

It is interesting to note that the multivariable analyses for OS in the TS and ESR, and LCSS in the ESR populations yielded similar results to the multivariable analyses for OS in our companion manuscript containing two different lung cancer populations (all patients presenting with NSCLC and those presenting with Stage IV disease). In all four lung cancer populations in both manuscripts, well-established risk factors (12, 13) for OS and LCSS were noted in all multivariable analyses including tumor size, stage, differentiation, gender, age, and *t*-stage. After adjustment for histolopathologic, gender, age, treatment, and marital variables, all ethnicities in all analyses had similar or significantly better OS and LCSS (ESR group only) compared to the White group. Adenocarcinoma was uniformly associated with a better OS. A consistently lower OS and LCSS were noted for all four lung cancer populations in Greater Georgia, Louisiana, and Kentucky. Similarly, patients in California and Iowa had poorer outcomes except for OS in the Stage IV population in California and OS in the ESR group in Iowa. The reason for the consistently poor outcomes across all stages and presentations in these registries is currently not known, but we believe that the number physician per 100,000 may be a factor because all five states rank in the bottom half of states in terms of the density of total active physicians as well as primary care physicians (14). Of interest, the highly significantly survival decrement (*p* < 0.0001) for tumor location in the mainstem bronchi in the companion manuscript was less significant in the surgical patients where only the right mainstem (*p* = 0.01) remained significant for OS in the TS group. There was no OS or LCSS decrement noted in the ESR population for the mainstem bronchi location. However, there was only a small number of tumors associated with the mainstem bronchi (*N* = 30) in the ESR group. We hypothesize that surgery neutralizes the effects of mainstem bronchi locations because this modality effectively eradicates a location that can cause obstructive pneumonias in a compromised patient group. Interestingly, although the companion paper noted that both lower lobe locations were noted to be associated with decreased OS, only the right lower lobe location was noted to be associated with worse OS in the surgical patients. The association of the lower lobes with worse outcomes has been noted in other investigations (15, 16). Our analysis demonstrates that the worse OS survival in patients having tumor located in the right lower lobe may be due to an increased risk of nodal involvement. Prognosis in all lung cancer populations was improved by being married, not having Medicaid, and being insured, but unlike the previous analysis, income was not correlated with LCSS and OS in the surgical patients in this investigation with the exception of borderline worse of OS in the TS population for those individuals with a median household income of <$50,000 (*p* = 0.0457). In addition, all lung cancer populations were noted to have a general improvement in OS during the years of this study. The improvement in the surgical populations may have been due to variables that are not contained within SEER such as improved staging, increased use of chemotherapy, and better post-operative care. However, the improved OS in the ESR group would argue against the increased use of adjuvant therapy because chemotherapy would be less likely to be used in this group (17, 18). Likewise, it may be argued that better post-operative care did not contribute to the better OS of the TS population because the 90-day mortality did not improve during the years of this study.

This manuscript was able to assess some treatment-related factors because SEER-18 does contain some variables related to radiation and surgery. Patients receiving pre-operative radiation were excluded because it was felt that this treatment could obscure/improve histolopathologic variables. Because SEER-18 does not contain information pertaining to chemotherapeutic treatment, we deliberately decided to separately assess a surgical sub-group of patients with tumors 4 cm or less without nodal involvement because these patients would be unlikely to receive chemotherapy (17, 18). Furthermore, we decided to investigate LCSS as well in this group of early-stage patients because of their relatively high likelihood of surviving lung cancer and possibly succumbing to other smoking-related causes. Worse OS and LCSS were consistently noted after a pneumonectomy despite multivariable analyses that accounted for histopathologic, patient, and tumor location variables. The adverse survival of patients undergoing a pneumonectomy was identified in recent retrospective study that demonstrated that that the lower survival may be due to an increased risk of distal metastases (19). Although the immune effects of a larger lung cancer procedures such as pneumonectomy as compared to (bi)lobectomy and sub-lobar resections is not known, it has been shown that transthoracic surgery for esophageal cancer as compared to smaller and less invasive surgical procedures (gastrectomy for cancer and cholecystomy for benign gallstones) has been associated with a transient immunosuppression (increased T-cell apoptosis and decreased T-cell cytokine production) during post-operative days 1–3 (20). Interestingly, a different research group noted that both transhiatal and transthoracic esophagectomies were associated with reduced TH1-type cytokine production on post-operative day 1, but depression of Th2-type cytokine was more profound with the latter procedure (21). In both surgical populations, the number of nodes examined was strongly correlated with OS and LCSS and was similarly noted in a past SEER analysis (22). The better outcomes associated with an increasing number of nodes examined may be due to the removal of microscopic disease that may or may not be recognized (especially in the ESR group) by routine pathologic methods (23), but because there is no OS with mediastinal lympadenectomy as compared to nodal sampling (24), one might infer that the beneficial effects of lymph node examination may be due to upstaging cancers that would otherwise be classified as node negative. Post-operative radiation was associated with poorer OS and LCSS. Although past retrospective analyses have demonstrated a possible survival benefit for radiation therapy in patients with N2 disease (25, 26), others have not (27). However, there has been general agreement that post-operative radiation results in a survival decrement in patients with N0 and N1 disease (25, 26). A recent retrospective investigation demonstrated that there was an OS benefit for post-operative radiation therapy for patients who experience a positive resection margin for all nodal stages (28). We would assume that the patients who receive post-operative radiation therapy for nodal stages N0–N1 during the years of our study had a positive margin, but SEER does not have information concerning margin status, and our results show a strongly negative effect of radiation on OS and LCSS in the surgical patients. Although there may be negative selection factors (i.e., positive margin, lymphatic, and/or vascular invasion) in the patients receiving radiation, it may be that radiation therapy has no efficacy and could possibly only have deleterious effects in the post-operative setting, especially in those with N0–N1 disease.

The MVA for 90-day OS revealed that mortality was not related to ethnicity, but was significantly correlated with single/unmarried partner status, Medicaid or unknown insurance, and income. Nevertheless, several known histopathologic and patient prognostic factors associated with aggressive disease/poor outcomes predicted 90-day mortality included increasing patient age, male sex, tumor differentiation, stage, and non-adenocarcinoma histology and suggest that aggressive tumor spread and/or understaging at the time of resection may be the reasons for poor early survival. However, because financial and partnership variables did affect 90-day mortality, one may conclude that patients may be able to improve their short-term survival by better economic and emotional support. Of interest, even after accounting for histopathologic characteristics, tumor locations in the right mainstem bronchus and right lower lobe were associated with a decrement in OS. We hypothesize that operative complications associated with these locations may be a reason why these sites adversely affect OS in the TS and ESR populations. Treatment-related factors related to an increased mortality included the performance of a pneumonectomy and less nodes examined. We decided to include radiation in this analysis because we felt that radiation could possible result in an increased early mortality. Interestingly, radiation was strongly associated with an improved 90-day survival which may be due to patient selection factors which are not acknowledged by SEER including a better ECOG performance status, less co-morbidities, and lower risk of immediate post-operative infections. Early mortality did not improve during the years of our investigation suggesting that post-operative care was not associated with the improved outcomes in surgical patients.

The decision to assess tumors generally considered eligible for a sub-lobar resection (T1–T2 tumors <2 cm in size) was made in order to assess which patients would benefit from a lymphadenectomy. Not surprisingly, nodal positivity was associated with known prognostic factors including advanced age, male sex, *t*-stage, aggressive histologies (adenosquamous, large cell, and squamous carcinomas), and tumor differentiation. Importantly, it should be noted that ethnicity was not associated with an increased risk of having positive nodes. Although income was not associated with nodal positivity, not being insured and not being married were both strongly associated with having node involvement. Because this analysis revealed that the right lower lobe location was associated with positive nodes, we believe that this may be a reason why this location is associated with a lower OS in both the TS and ESR populations.

We originally performed this analysis to assess the effects of the presentation and outcome differences by ethnicity as compared to Whites in patients undergoing surgical resection for lung cancer. In comparison to White patients, OS, LCSS, and 90-day mortality were similar or better in all ethnic groups for all three analyses. Median household income was largely not associated with OS or LCSS in the TS and ESR patients, but was strongly associated with 90-day mortality. Because this variable was assigned to patients based upon the median county income, we assume this variable may have adversely affected 90-day survival due to the hospital care received in more wealthy and less affluent areas. Of importance, Medicaid insurance and not being married were associated with lower OS and LCSS as well as an increased risk of 90-day mortality. We feel that not Medicaid insurance is more likely to represent an individual’s economic status and demonstrates the importance of having insurance. However, of great interest, is that having Medicaid and not being married are factors that were also associated with an increased risk of nodal involvement. This suggests that economic and psychological factors can possibly be associated with lung cancer biology. Lower socioeconomic status may affect tumor biology through poor nutrition (29). Recently, it was noted that unmarried lung cancer patients had a greater incidence of depression, less social support, and a survival decrement (30), and that the survival decrement noted in patients with new-onset or persistent depression may be more so in early-stage (Stages I–II) than in patients with more advanced stages (31). We feel that our results suggest that the economic effects of not having insurance and not being married are associated with real changes in tumor biology and aggressiveness.

It should be noted that the SEER database lacks may variables that would have been useful for our analysis including smoking history, body mass index, ECOG performance status, lymphatic and/or vascular invasion, patient co-morbidities, chemotherapy administration, type of surgical procedure (i.e., VATS, robotic surgery, and traditional thoracotomy), radiation dose, and radiation field arrangement. However, we have no reasons to think that any of these variables would have influenced our outcomes because we could account for median household income, type of insurance, and most major histopathologic variables.

In summary, the main purpose of our investigation was to assess difference in outcomes (OS and 30/90 day mortality), presentation, and treatment in nine different ethnic groups who underwent surgical resection of NSCLC. As a secondary aim, we also wanted to assess whether tumor biology (nodal involvement) varied by ethnicity. Even in the analyses that were not adjusted for treatment, histopathologic, patient, and marital factors; Blacks and Hispanics had the same OS and LCSS as the White group. We did not find disparities due to ethnicity in patients undergoing surgical resection for NSCLC, but noted that the disparities may be due to having Medicaid insurance and not being married. Because having Medicaid insurance and not being married were associated with lower OS, LCSS and 90-day OS as well as nodal positivity, we feel that economic and psychosocial variables may play a role in the biological aggressiveness of early-stage lung cancer patients undergoing resection in addition to standard histopathologic and treatment variables. Although marriage was equally as important as socioeconomic factors in our assessment, a study from an earlier time period (1989–2003) suggested that lower socioeconomic status was an independent prognostic factor, but marriage was note (32). However, this past investigation by Ou et al. also noted that race was not a prognostic factor in multivariate modeling.

## Conclusion

In TS and ESR populations, OS was not different in the two largest ethnic groups (Black, Hispanic) as compared to Whites, but was related to single/divorced status, medicaid insurance, and income (TS population only). Nodal positivity was associated with patients who did not have a married partner or insurance suggesting that these factors may impact disease biology. Economic and psychosocial variables may play a role in survival of early-stage lung cancer in addition to standard histopathologic and treatment variables.

## Author Contributions

Writing, editing, and manuscript approval—JV, KM, RV, MD, JF, NR, TF, PR, WW, DM, KU, JB, and PO. Data acquisition—JV, KM, and RV. Data analysis—JV, KM, RV, MD, and JF.

## Conflict of Interest Statement

The authors declare that the research was conducted in the absence of any commercial or financial relationships that could be construed as a potential conflict of interest.
